# Prediction of Long-Term Mortality by Preoperative Health-Related Quality-of-Life in Elderly Onco-Surgical Patients

**DOI:** 10.1371/journal.pone.0085456

**Published:** 2014-01-20

**Authors:** Maren Schmidt, Bruno Neuner, Andrea Kindler, Kathrin Scholtz, Rahel Eckardt, Peter Neuhaus, Claudia Spies

**Affiliations:** 1 Department of Anesthesiology and Intensive Care Medicine, Campus Charité Mitte and Campus Virchow-Klinikum, Charité-University Medicine Berlin, Berlin, Germany; 2 Charité Research Group on Geriatrics, Charité-University Medicine Berlin, Berlin, Germany; 3 Department of General, Visceral, and Transplantation Surgery, Charité Campus Virchow, Charité-University Medicine Berlin, Berlin, Germany; The University of Hong Kong, Hong Kong

## Abstract

**Objective:**

Aim of this study was to evaluate the association between preoperative health-related quality of life (HRQoL) and mortality in a cohort of elderly patients (>65 years) with gastrointestinal, gynecological and genitourinary carcinomas.

**Design:**

Prospective cohort pilot study.

**Setting:**

Tertiary university hospital in Germany.

**Patients:**

Between June 2008 and July 2010 and after ethical committee approval and written informed consent, 126 patients scheduled for onco-surgery were included. Prior to surgery as well as 3 and 12 months postoperatively all participants completed the EORTC-QLQ-C30 questionnaire (measuring self-reported health-related quality of life). Additionally, demographic and clinical data including the Mini Mental State Examination (MMSE) were collected. Surgery and anesthesia were conducted according to the standard operating procedures. Primary endpoint was the cumulative mortality rate over 12 months after one year. Changes in Quality of life were considered as secondary outcome.

**Results:**

Mortality after one year was 28%. In univariable and multivariable logistic regression analysis baseline HRQoL self-reported cognitive function (OR per point: 0.98; CI 95% 0.96–0.99; p = 0.024) and higher symptom burden for appetite loss (per point: OR 1.02; CI 95% 1.00–1.03; p = 0.014) were predictive for long-term mortality. Additionally the MMSE as an objective measure of cognitive impairment (per point: OR 0.69; CI 95% 0.51–0.96; p = 0.026) as well as severity of surgery (OR 0.31; CI 95% 0.11–0.93; p = 0.036) were predictive for long-term mortality. Global health status 12 months after surgery was comparable to the baseline levels in survivors despite moderate impairments in other domains.

**Conclusion:**

This study showed that objective and self-reported cognitive functioning together with appetite loss were prognostic for mortality in elderly cancer patients. In addition, impaired cognitive dysfunction and severity of surgery were predictive for one-year mortality whereas in this selected population scheduled for surgery age, gender, cancer site and metastases were not.

## Introduction

The incidence of most solid tumors increases with age. Over 50% of solid tumors and 80% of cancer deaths occur in patients older than 65 years [1], [2]. Contrary to better survival rates in younger patients, mortality of elder cancer patient remains high in spite of enhancements in conservative and surgical cancer therapies [1]–[3]. On one hand, comorbid conditions and progressive reduction of organ functions are potential reasons why elderly patients less likely tolerate chemotherapy and surgery. On the other hand, elderly patients are excluded from standard therapy often only due to their chronological age [1], [4], [5].

Equal important than survival itself in elderly patients are function preservation and maintenance of quality of life [3], [6]. Incorporating health-related Quality-of-Life (HRQoL) as a treatment outcome parameter should, therefore, be established in oncology [7]. The self-reported health status of patients is considered as important as traditional outcome parameters like overall survival and recurrence–free survival. Reasons to extend the traditional endpoints as survival, disease-free survival etc. are numerous [8]. Cancer and the consequences of cancer surgery may have an important impact on a patient’s quality of life [9] and lead to overall poor quality of life [8] through pain, fatigue, depression and distress [10], [11].

Evidence on quality-of-life outcomes could be used to inform patients about their expected recovery as well as about treatment effects and thus help them in making informed treatment decisions together with their relatives [12], [13]. Furthermore, there is evidence that baseline assessment of HRQoL provides prognostic information on survival in various types of cancer patients such as those with colorectal, esophageal, breast, prostate and lung cancer [7], [14], [15]. HRQoL has been shown to be more sensitive about the severity of cancer than conventional prognostic indices or as assessment by a physician [14], [16]. In a review by Gotay et al [17], in 36 out of 39 studies at least one of the HRQoL domains was significant predictive for survival. However, results have been inconclusive. Studies have investigated patients with different disease sites, with different advanced stages and used different instruments for assessment of HRQoL making it overall difficult to compare the published results.

It is known, that many domains of HRQoL are age-dependent. Still, there is a lack of HRQOL studies in elderly patients [3], [18]. Hence, aims of this study were to determine if and to what extent preoperative quality of life scores predict one-year mortality after major cancer surgery in elderly cancer patients and to describe changes in the quality of life in elderly patients from the preoperative baseline examination up to 12 months postoperatively. This study was designed as a pilot study to provide a basis for a multicenter interventional study and to test the feasibility of the recruitment and the acceptability of the questioning in elderly onco-surgical patients.

## Methods

### Ethics Statement

The study was conducted in compliance with the Helsinki declaration. The institutional Review Board of Charité-Universitaetsmedizin Berlin approved the study (EA 2/103/07). All participants gave their written informed consent.

### Study Design

Prospective, observatory cohort pilot study at a tertiary university hospital.

### Patients

From June 2008 to July 2010, all patients older than 65 years scheduled for surgery for gastrointestinal, genitourinary or gynecologic cancer were screened of eligibility.

Patients were eligible if they were able to understand or read German language, reached a Mini Mental Score (MMSE) of 23 points or higher and were able to provide a written informed consent.

**Figure 1:**
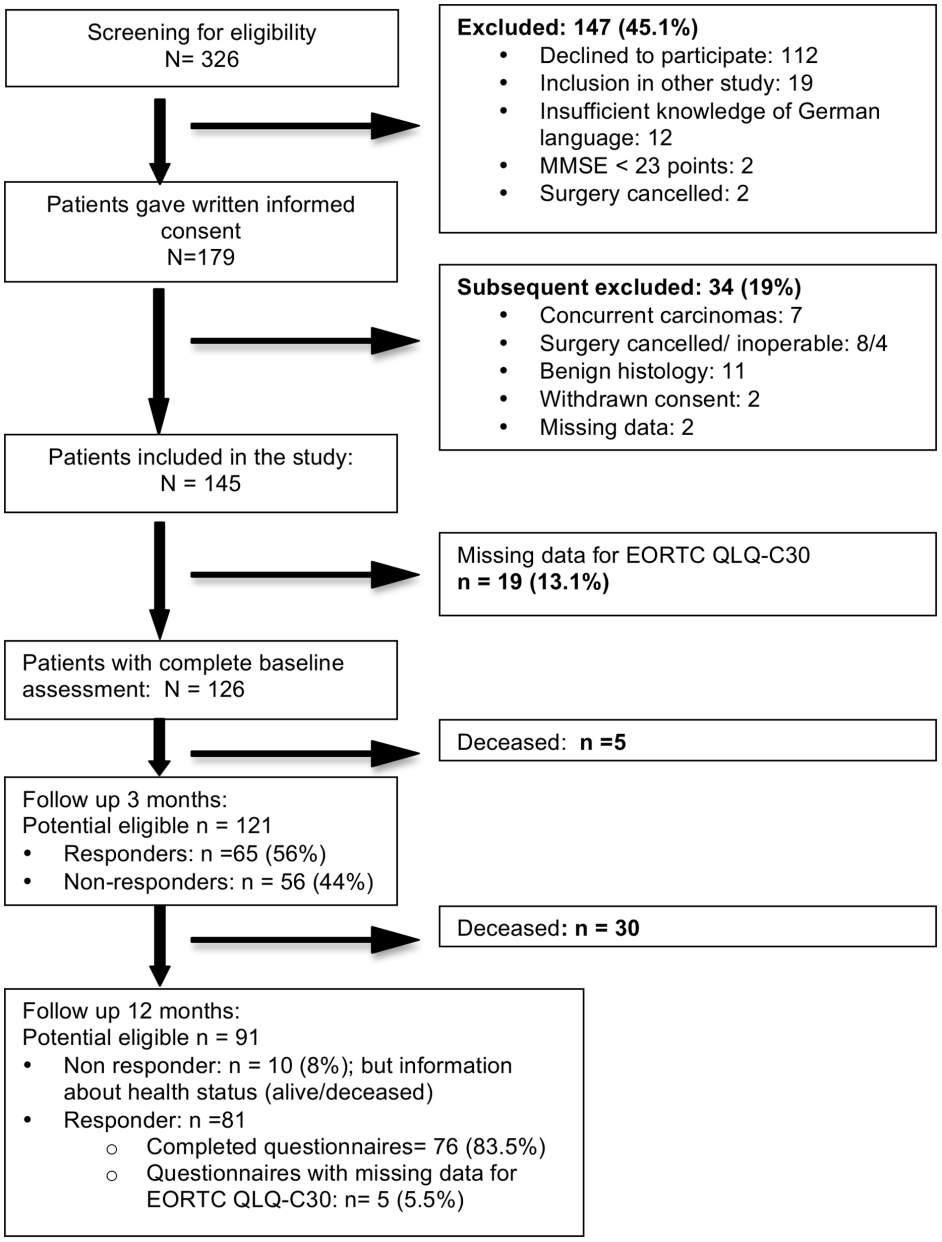
Flow chart of the study population.

Exclusion criteria were age under 65 years, two or more concurrent carcinomas, emergency surgery, and participation in another trial and insufficient knowledge of the German language. Patients unable to give informed consent were not included. (Fig. 1).

### Perioperative Treatment

Anesthesia, surgery and perioperative treatment were carried out according to the standard operating procedures in our university-hospital [19].

### Data Collection

#### Health related quality of life measurement

We obtained HRQOL scores from a validated questionnaire, the European Organisation for Research and Treatment of Cancer 30-Item Core Quality of Life Questionnaire, version 3.0 (EORTC QLQ-C30). Questionnaires were self-completed by patients within 3 days prior to surgery as well as, using mailed questionnaires, at 3 and 12 months after surgery, respectively [20].

The EORTC QLQ-C30 questionnaire is a 30–item questionnaire incorporating nine multiple-item scales and six single items. Multiple-item scales consisted of global health scale, three symptom scales (fatigue, nausea and vomiting, pain) and five functional domains (physical, role, cognitive, emotional, and social). Six single items scales include appetite loss, dyspnea, sleep disturbance, constipation, diarrhea and financial impact. All scales were answered using a 4-point Likert scale except for the global health question, which uses a 7-point Likert scale. All QOL scores were linearly transformed to a score from 0 to 100 and missing items were handled as in the manual described [21]. Higher scores in symptom scales reflect higher symptom burden, whereas higher scores in functional scales and in global quality of life reflect higher levels of functioning and a higher quality of life, respectively. A difference of 5–10 point in the scores represents a small change, 10–20 points a moderate change, and more than 20 points a large change in domains of QoL [22]. The EORTC QLQ-C30 is a reliable and valid instrument for assessing quality of life in cancer patients [14], [20]. To compare data for this disease specific questionnaire to population-based norms, Schwarz and Hinz published age- and sex-adjusted reference data for the EORTC QLQ-C30 in the general German population in 2001 [23].

The reliability coefficients (as measured by Cronbach’s α) in this study population were high and similar in the global health status (α = 0.879), physical functioning (α = 0.822), role functioning (α = 0.833), and social functioning (α = 0.811). The coefficients for emotional functioning (α = 0.554), cognitive functioning (α = 0.517), pain (α = 0.584), nausea and vomiting (α = 0.627), fatigue (α = 0.693) were somewhat lower [24].

Demographic and clinical data were recorded at baseline. The collected data included age, gender, marital and sociodemographic status, body mass index (BMI), co-morbidities documented as Charlson Comorbidity Score [25], ECOG performance status [26], preoperative risk assessment according to the classification of the American Society of Anesthesiologists (ASA) [27], severity of surgery measured by the Physiological and Operative Severity Scoring system for enUmeration of Mortality and morbidity (POSSUM) [28], cancer site and metastases. Intra-operative parameter encompassed the duration of surgery, type of anesthesia and length of anesthesia. Additionally the duration of hospitalization, morbidity and in-hospital mortality was recorded. Duration of hospitalization was defined as time in hospital after surgery. Perioperative complications and morbidity were classified according to the Clavien-Scale [29]. Postoperative complications of Clavien grades 3 to 5 were defined as major complications [30].

Cognitive measures included the Folstein Mini-Mental State Examination (MMSE) [31]. The MMSE is a broadly used bedside test for screening for cognitive impairment. The test consists of 30 items sampling 7 categories: orientation, registration, attention and concentration, memory, language, calculation and visual construction. The maximum score is 30 points. A higher score represents a better cognitive function while a score lower than 24 points indicates cognitive impairment [31]. For a community-dwelling population over 65 years the mean score is 27 points [32]. Patients were tested before inclusion in the study.

Additionally, the participants filled in the Geriatric Depression Scale (GDS) at the baseline visit [33].

#### Follow up

The patients were followed up for 1 year. All patients were contacted via mail three and 12 months after surgery. Survival status 12 months after surgery was recorded for all patients. The participants got questionnaires including the EORTC QLQ C30 by mail. If patients did not answer within two weeks, they were contacted by telephone. In case of patients lost to follow-up, the family doctor was asked about patients’ survival status. The permission to contact the general practitioner was obtained from the participants at the baseline visit prior to operation.

### Statistical Analysis

Categorical variables are presented as numbers and percentages. Continuous variables are presented as mean and standard deviation (SD) and, when not normally distributed as median (interquartile range, IQR). For categorical variables differences between two independent groups respectively more than two independent variables were examined using the *X* ^2^–test respectively the Cochrane-Armitage test for trend. Differences between two independent groups in normally distributed continuous variables were evaluated using the students t-test respectively the Mann-Whitney U test for continuous non-normally distributed variables.

Patients’ mortality was the primary end point and defined as cumulative mortality rate over 12 month (long-term mortality). The relationship between baseline QoL and long-term mortality was analyzed using univariable and multivariable logistic regression analysis adjusted for relevant covariates.

For univariable logistic regression analysis all domains of the EORTC-QLQ C-30 were tested with long-term mortality as dependent variable. Furthermore clinical variables like age, gender, cancer site, presence of metastases, ECOG performance status (dichotomized 0 vs. <0), ASA state (dichotomized I+II vs. III), severity of surgery (dichotomized mild/moderate vs. severe), Mini Mental State, GDS (dichotomized depression vs. no depression), marital status (alone vs. married), BMI, Charlson Comorbidity Score and complications (no vs. yes) were tested in univariable logistic regression analysis against long-term mortality.

Those variables found to predict long-term mortality were then included in multivariable analyses, which were additionally adjusted for age, gender and distant metastases to identify independent predictors for long-term mortality. The effect of QoL parameters on patients’ long-term mortality was expressed as odds ratio (OR) with the corresponding 95% confidence intervals (CIs).

The secondary outcome, i.e. change in QoL scores over 12 months, was evaluated comparing differences in baseline HRQoL scores and HRQoL scores at 12 months. We used the t-test for paired samples. Changes of 10 or more points on a zero-to-100 scale are considered clinically relevant [22].

Statistical significance was defined as p<0.05. All statistical tests were two-sided. All statistical analyses were performed using SPSS software (IBM® SPSS® Statistics20; SPSS, Inc., Chicago, IL, USA).

## Results

### Patient Recruitment and Follow Up

The outline of patient recruitment and follow-up is shown in Figure 1. The final sample consisted of 126 patients (see Fig. 1). After 3 and 12 months questionnaires were sent to the patients. We received answers and information about mortality in 116 patients (92%): 35 patients died (27%) and 81 (64, 3%) questionnaires were sent back to us. From these questionnaires 5 were incomplete. From 10 (8%) patients we received no personal answer, but we got information on survival via their general practitioner.

### Baseline Characteristics

The sociodemographic and clinical characteristics of survivors and non-survivors are listed in Table 1. The mean age of all patients was 72±5.6 years. There were more women (55.6%) than men (44.4%). Duration of hospitalization ranged from 2 to 144 days, with a median of 13 days.

**Table 1:** Patients characteristic – Sociodemographic and clinical variables, stratified for Non-Survivors/Survivors.

	All	Non-Survivors	Survivors	p
	N = 126 (100%)	N = 35 (27.8%)	N = 91 (72.2%)	
**Age in years,**				
Mean (SD)	72 (5.6)	73 (6.1)	71.8 (5.4)	0.302^2^
Min - Max	65–91	65–91	65–88	
**Gender,** Female	70 (55. 6%)	18 (51.4%)	52 (57.1%)	0.563^3^
**BMI,** Mean (SD)	26.7 (4.9)	26 (4)	27 (5.2)	0.444^2^
**MMSE,** Median (IQR)	29 (28; 29)	28.5 (27; 29)	29 (28; 30)	0.079^2^
**Marital status:**				0.573^3^
Single	12 (9.5%)	4 (11.4%)	8 (8.8%)	
Married	76 (60.3%)	23 (65.7%)	53 (58.2%)	
Divorced/widowed	3/35 (30.2%)	0/8 (22.9%)	3/27 (33%)	
**Cohabitation:**				0.784^3^
Living alone	34 (27%)	8 (22.9%)	26 (28.6%)	
With family	92 (73%)	27 (77.2%)	65 (71.5%)	
**Level of education**				0.107^3^
< Compulsory school	2 (1.6%)	2 (5.7%)	0 (0%)	
Compulsory school	46 (36.5%)	11 (31.4%)	35 (38.5%)	
> Compulsory school	61 48.4%)	16 (45.7%)	45 (49.5%)	
Missing	17 (13.5%)	6 (17.1%)	11 (12.1%)	
**ASA**				0.804^4^
I	10 (7.9%)	2 (5.7%)	8 (8.8%)	
II	62 (49.2%)	18 (51.4%)	44 (48.4%)	
III	55 (42.9%)	15 (42.9%)	39 (42.9%)	
**Severity of surgery** **				*0.024* 4
I–low	6 (4.8%)	0 (0%)	6 (6.6%)	
II–middle	33 (26.2%)	6 (17.1%)	27 (29.7%)	
III–high	87 (69.0%)	29 (82.9%)	58 (63.7%)	
**Complications (%)**				
Any complication	84 (66.7%)	27 (77.1%)	57 (63.3%)	0.140^3^
Minor complications	47 (37.3%)	11 (31.4%)	36 (39.6%)	*0.040* 4
Major complications	37 (29.4%)	16 (45.7%)	22 (24.2%)	
**Comorbidity Charlson:**				
Median (IQR)	4 (2; 6)	4 (3; 7)	4 (2; 6)	0.248^2^
Min–Max	2–10	2–9	2–10	
**ECOG Performance status**				*0.019* 4
0	72 (57.1%)	16 (44.4%)	56 (61.5%)	
1	44 (34.9%)	14 (38.9%)	31 (34.1%)	
2	7 (5.6%)	4 (11.1%)	3 (3.3%)	
3	3 (2.4%)	2 (5.6%)	1 (1.1%)	
4+5	0 (0%)	0 (0%)	0 (0%)	
**Metastases**	70 (55.6%)	24 (68.6%)	46 (50.5%)	0.068^3^
**Length of postoperative stay (days)**				*0.005* ^2^
Median (IQR)	13 (9; 19)	17 (11; 24)	12 (9; 16)	
Min–Max	2–144	2–144	2–66	
**Geriatric depression scale**				0.071^3^
No depression (0–5 pts)	116 (92.1%)	32 (91.4%)	84 (92.3%)	
Moderate (6–10 points)	8 (6.3%)	1 (2.9%)	7 (7.7%)	
Severe (11–15 points)	2 (1.6%)	2 (5.7%)	0 (0%)	
**Primary cancer site**				0.345^3^
Upper GI	51 (40.5%)	18 (51.4%)	33 (36.3%)	
Colorectal	33 (26.2%)	6 (17.1%)	27 (29.7%)	
Gynecological	6 (4.8%)	10 (28.6%)	26 (28.6%)	
Urogenitary	36 (28.6%)	1 (2.9%)	5 (5.5%)	

^2^Mann – Whitney –U-Test,

^3^Χ^2^ test,

4 Cochran-Armitage trend test;

** Measured by the Physiological and Operative Severity Scoring system for enUmeration of Mortality and morbidity (POSSUM) [28].

SD: standard deviation, IQR: interquartile range, BMI: body mass index, GDS: geriatric depression scale; GI: Gastrointestinal, ASA: American Society of Anesthesiologists.

Most of the patients had a performance status of 1 or better (92%). Metastases were present in 55.6% of all patients. Cancer site was upper and lower gastrointestinary tract (40.5% and 26.2%), urogenitary (4.8%) or gynecological (28.6%).

### Mortality and Morbidity

The 1-year mortality rate was 27%. Two (1.5%) patients died in hospital but later than 30 days after surgery. Another three (2.3%) patients died within the first three months. Overall, 38 (30.2%) patients had major complications and 47 (37.3%) minor postoperative complications according to the Clavien Scale [29].

### Health-related Quality of Life

**Figure 2:**
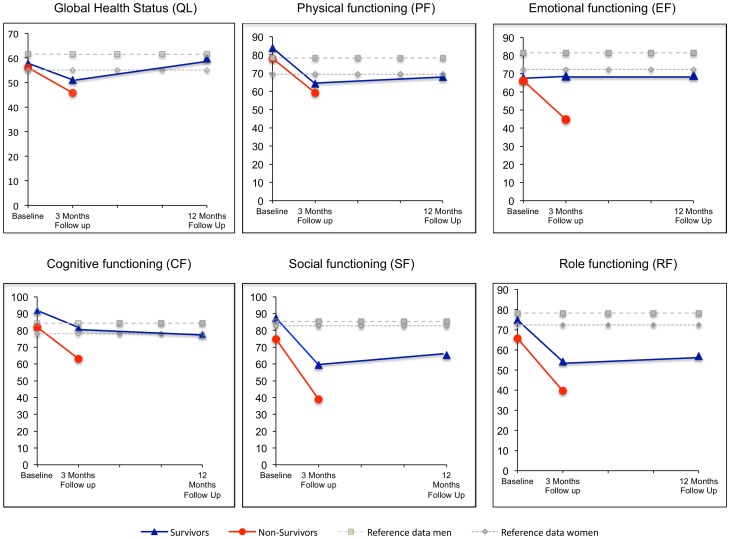
Changing of functional domains of Health Related Quality of life from preoperative baseline to 12 months follow up. The mean scores of the EORTC QLQ-C30 Global health score and the functional domains for survivors, non-survivors (data baseline and follow up after 3 months), and the reference values for women and men for the same-aged German population are shown. Higher scores represent better function and higher global health. In the baseline, non-survivors had significant lower self-reported cognitive function and social function at baseline than survivors (p = 0.020 and p = 0.013). There was no significant difference in global health status or in the remaining functional scales between survivors and non-survivors. The mean scores of global health and all functional scales fell 3 months after surgery. Global health and emotional functioning recovered from the decline in 12 months with a small, not significant improvement compared to baseline, whereas physical, cognitive, social and role functioning were significant worse than before surgery (p<0.001). For physical, cognitive and role functioning the decline one year after surgery was moderate, whereas social functioning showed a large decrease between baseline test and the 12 months follow up.

**Figure 3:**
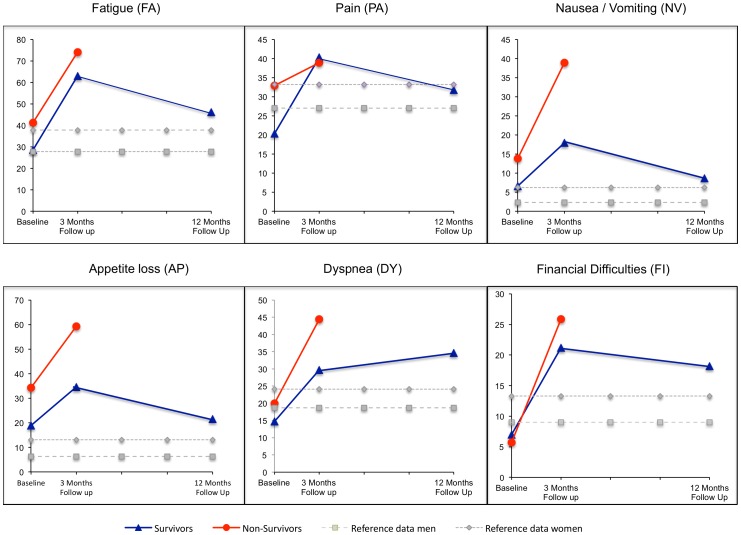
Changing of symptom related Health Related Quality of life–domains from preoperative baseline to 12 months follow up. The mean scores of the EORTC QLQ-C30 symptoms domains for survivors, non-survivors (data baseline and follow up after 3 months) and the reference values for women and men for the same-aged German population are shown. Higher scores represent higher symptom burden. The burden of appetite loss in the baseline was significant higher in non-survivors (p = 0.008) whereas the remaining symptom scales were not significant different between both groups in baseline assessment. The symptom burden increased in all domains 3 months after surgery. In survivors, one year after surgery, appetite loss and nausea and vomiting were similar to baseline levels, whereas fatigue (p<0.001), pain (p = 0.015), dyspnea (p<0.001), and financial difficulties (p = 0.001) were worse compared to baseline but improved in comparison to the 3 months follow up. The increase in symptom burden was moderate (10–20 points increase).

The mean scores of the EORTC QLQ-C30 domains for survivors and non-survivors are shown in Figures 2 and 3.

Non-survivors had significant lower self-reported cognitive function respectively social function at baseline than survivors (p = 0.020 respectively p = 0.013). In the symptom scales the burden of appetite loss was significant higher in non-survivors (p = 0.008). The differences in the mean scores were classified as moderate according to Osoba et al [22]. There was no significant difference in baseline global health status or in physical functioning between survivors respectively non-survivors (p = 0.438 respectively p = 0.412). Emotional and role functioning as well as the remaining symptom scales were not significant different between both groups.

### Prediction of Survival

Overall mortality after one year was 27%. In univariable logistic regression analysis (Table 2), the HRQoL subdomains cognitive functioning (p = 0.020), social functioning (p = 0.033), fatigue (p = 0.043), pain (p = 0.042) and appetite loss (p = 0.033) were predictive for mortality. Amongst the included sociodemographic and biomedical variables severity of surgery (p = 0.043) and the MMSE (p = 0.013) predicted mortality either. ASA state (p = 0.557), gender (p = 0.563), age (p = 0.239), performance state (p = 0.110), comorbidities (p = 0.373) perioperative complications (p = 0.144), metastases (p = 0.072) and cancer site (p = 0.759) had no prognostic impact in univariable analyses. Postoperative HRQoL values after 3 months had no significant influence on survival after 12 months.

**Table 2:** Univariable logistic regression analyses of long-term mortality for sociodemographic, clinical, and Health-related Quality of Life Data (HRQOL) Data.

*Sociodemographic and* *clinical variables*	OR (CI 95%)	P
Age	1.04 (0.97–1.11)	0.239
**Severity of operation** ** **:**		
**Mild/moderate vs. severe**	**0.36 (0.13–0.96)**	**0.043**
Metastases (no vs. yes)	0.46 (0.20–1.07)	0.071
ASA:		
II vs I	1.64 (0.32–8.47)	0.557
III vs I	1.54 (0.29–8.09)	0.611
Gender (men vs. women)	0.79 (0.36–1.74)	0.563
Cancer site:		
Abdominal vs. gynecological/urogenitary	1.13 (0.49–2.6)	0.779
**Mini Mental State (MMSE)** per point	**0.69 (0.53–0.93)**	**0.013**
Marital Status (alone vs. married)	0.73 (0.32–1.6)	0.443
Geriatric Depression Score (GDS):		
(No depression vs. depression)	0.89 (0.22–3.65)	0.870
Body Mass Index (BMI) per unit	0.96 (0.89–1.05)	0.377
Charlson Comorbidity Score (CCS) per point	1.08 (0.91–1.29)	0.373
Performance State (impaired vs. good)	1.90 (0.86–4.18)	0.110
Complications (no vs. yes)	1.95 (0.79–4.80)	0.144
***HRQOL variables*** ***		
Global health status (QL)^3^	0.99 (0.98–1.01)	0.681
Physical Functioning (PF)^3^	0.99 (0.97–1.01)	0.160
Emotional functioning (EF)^3^	0.99 (0.98–1.01)	0.803
**Cognitive functioning (CF)** ^3^	**0.98 (0.96–0.99)**	**0.020**
**Social functioning (SF)** ^3^	**0.99 (0.97–0.99)**	**0.031**
Role functioning (RF)^3^	0.99 (0.98–1.00)	0.175
**Fatigue (FA)** 4	**1.01 (1.00–1.03)**	**0.043**
**Pain (PA)** 4	**1.01 (1.00–1.03)**	**0.042**
Nausea and Vomiting (NV)^4^	1.02 (0.99–1.04)	0.081
Dyspnea (DY)^4^	1.01 (0.99–1.02)	0.362
Insomnia (SL)^4^	0.99 (0.99–1.01)	0.527
**Appetite Loss (AP)** 4	**1.01 (1.00–1.02)**	**0.033**
Constipation (CO)^4^	1.00 (0.99–1.01)	0.873
Diarrhea (DI)^4^	1.00 (0.98–1.03)	0.768
Financial difficulties (FI)^4^	0.99 (0.98–1.02)	0.739

OR = Odds ratio; CI = Confidence interval; p = p-value; ASA = American Society of Anesthesiologists.

** Measured by the Physiological and Operative Severity Scoring system for enUmeration of Mortality and morbidity (POSSUM) [28].

*** EORTC QLQ-C30 Questionnaire; Continuous range;

^3^High scores represent better function;

4 High score represent worse symptoms.

In the multivariable logistic regression analysis (Table 3), variables significant in the univariable analysis (p<0.05) together with the variables age, gender and metastases were tested for prediction of mortality. Cognitive function (p = 0.024), appetite loss (p = 0.011), MMSE (p = 0.026) and severity of surgery (p = 0.036) were identified as prognostic factors for one-year mortality. Other quality of life domains or clinical parameters were not significant. Gender was not predictive for mortality: men versus women (OR = 0.46, 95%-CI: 0.18–1.17; p = 0.102).

**Table 3:** Multivariable regression analyses of mortality for sociodemographic, clinical, and Health-related Quality of Life Data (HRQOL) Data.

Demographic and clinical variables	OR (CI 95%)	P
Mini Mental State (MMSE),per point	0.69 (0.51–0.96)	**0.026**
Gender, men vs. women	0.46 (0.18–1.17)	0.102
Severity of operation:		
Mild/moderate vs. severe**	0.31 (0.11–0.93)	**0.036**
**HRQOL variables** ***		
Cognitive functioning (CF),per point	0.98 (0.96–0.99)	**0.024**
Appetite loss (AP), per point	1.02 (1.00–1.03)	**0.011**

Adjusted for age, gender and distant metastases.

OR: Odds ratio; CI = Confidence interval; p = p-value.

** Measured by the Physiological and Operative Severity Scoring system for enUmeration of Mortality and morbidity (POSSUM) [28].

*** Continuous range; EORTC QLQ-C30 Questionnaire.

### Trajectory of HRQoL Data

Reference data for German men and women older than 70 years are shown as shadowed values in figure 2 and 3 [23]. Due to the missing power of the study group differences between expected reference data and data of the study cohort are not tested for statistical differences. Global health scores for non-survivors and survivors are in the expected field, whereas survivors had higher self-reported physical and cognitive functioning than the expected average reference population. Both groups had lower values in emotional functioning and non-survivors reported worse social and role functioning than the reference population (Figure 2).

Symptom burden for appetite loss and nausea and vomiting were higher in both groups of the study population compared to the reference group. Survivors had lower pain burden whereas non-survivors reported worse fatigue symptoms than the average population (Figure 3).

The mean scores of global health and all functional scales fell 3 months after surgery (Figure 2). The decrease in global health and in emotional functioning was only small (<10 points decrease). Global health and emotional functioning recovered from the decline in 12 months with a small, not significant improvement compared to baseline (Figure 2), whereas physical, cognitive, social and role functioning were significant worse than before surgery (p<0.001). For physical, cognitive and role functioning the decline one year after surgery was moderate (10–20 point decrease/scale), whereas social functioning showed a large decrease between baseline test and the 12 months follow up. In the symptom scales (Figure 3), the symptom burden increased 3 months after surgery. One year after surgery, appetite loss, nausea and vomiting, insomnia were similar to baseline levels, whereas fatigue (p<0.001), pain (p = 0.015), dyspnea (p<0.001), diarrhea (p<0.001) and financial difficulties (p = 0.001) were worse compared to baseline but improved in comparison to the 3 months follow up. The increase in symptom burden was moderate (10–20 points increase).

## Discussion

In this study, self-reported cognitive impairment and objective measured limitation in the MMSE as well as self-reported appetite loss provided prognostic information for mortality up to one year after onco-surgery. Furthermore, the findings show that global health status 12 months after surgery is comparable to the baseline levels in survivors despite moderate impairments in other domains. There was no long lasting impact on global quality of life.

The population in the industrialized countries is aging. Physicians are faced with a growing number of older cancer patients where they need to agree on an operation or on a conservative treatment. Surgery is the state-of-the-art treatment for most solid tumors. Age disparities in therapy decision are well known for chemotherapy in elderly patients. For surgery there are only few studies looking at selection for surgery in elderly cancer patients [5], [34]. Especially elderly patients are denied to undergo onco-surgery due to the fear that they not survive and even in case of survival will not rehabilitate [1]. Hence, to identify prognostic factors in elderly cancer patients are necessary for treatment information and decision.

Around 3 out of 4 cancer patients survived the first year after operation. Considering the type of included tumors in this investigation mortality rates seem to be slightly better than published data [35], [36].

It is well known that in general impaired cognitive function in elderly persons is associated with increased mortality in community dwelled elderly adults as well as in elderly surgical and cancer patients [37]–[39].

Self – reported cognitive function was found as a predictive HRQoL domain in previous studies [17], [40], [41]. Blazeby et al. found impaired cognitive function to be predictive for 6 months mortality in patients with gastric and esophageal cancer [42]. In patients with breast cancer, a relationship between disturbed cognitive function (defined as forgetfulness, difficulties to concentrate) and disease-free survival was found [43]. Coates et al. found impairment in cognitive functioning predictive for survival in univariate analysis [41]. In a recently published study, cognitive function was not predictive in the baseline assessment in patient with cancer but predictive for survival in further progress of the disease [44].

The relationship between (self-reported) cognitive functioning and survival is not intuitively clear. Cognitive impairment is a known adverse effect of chemotherapy [45]–[47]. Subjective-assessed impairment in cognitive function might also be a sign of an advanced disease (e.g. chemotherapy) and therefore predictive for survival. Due to the study design, only patients with a MMSE of 23 points or better were included. Thus, participants had no manifest dementia.

The mean age of the cohort was 72 (SD: 5.6) years. Elderly patients are more vulnerable for postoperative delirium and postoperative cognitive dysfunction (POCD) than younger patients [48], [49]. Development of postoperative delirium or POCD could aggravate existing cognitive impairment with subsequent increased mortality [48], [49]. In this pilot study, we had assessed neither postoperative delirium nor postoperative cognitive dysfunction, so we could not confirm this hypothesis.

Appetite loss was predictive for survival, too. Appetite loss was an important independent predictor of survival in different cancer populations [14], [17]. Gotay et al. found appetite loss to be significant predictive in 10 of 39 studies [17]. One hypothesis is that this symptom is a very sensitive marker of patient wellbeing. Appetite loss is often a sign for advanced cancer with bowel restriction etc. and might be followed by malnutrition and weight loss, which has been described to influence survival negatively [17], [50]. In the main study, following this pilot study, a nutritional assessment and the estimation of body composition is included.

Several possible explanations for the demonstrated survival prediction may exist. An association with the tumor stage and tumor site could be hypothesized – especially in this heterogeneous study population. However, neither metastasis as a symptom for advanced cancer nor tumor site showed significant survival prediction.

HRQoL of patients could reflect an early perception of the severity of cancer in a more accurate way than conventional prognostic indices [14], [16]. Gaps between patient self perception and assessment by a physician are described in the literature [16]. Another hypothesis supposes that a better HRQoL score could have a positive effect on the disease process. However, there is a lack of clarity regarding the effect of psychosocial interventions on improvement of well-being and survival in cancer patients [7], [17], [51].

Global health status, physical function or performance status were no independent predictors for survival. These domains were often found to be predictive for short and long-term survival [14], [17]. HRQoL is eminent age-dependent and especially physical function is correlated with age [23], [52]. This correlation could be diminished in a cohort of patients older than 65 years. Further, physical status in prognostic studies could be a correlate for an early stage cancer and therefore not per se be predictive for survival [7]. Furthermore, the cohort of patients could be a positive selection of physical and mental fit patients. The baseline quality of life scores for physical function and cognitive function for survivors were higher than the calculated average score for the population in this age group. Even symptom burden like pain and fatigue, both typical side effects of cancer, were particularly better but at least not worse than the reference values. Studies have shown that older people with cancer have similar or better HRQOL when compared to non-cancer patients [53], [54]. Other studies have shown that increasing age is associated with decreasing health and HRQOL and differing expectation of HRQOL [55]. Compared to the calculated reference data for Germany [23], our patients had at baseline lower global life quality, but better functional scales than the reference population. Emotional functioning was the only exception with a lower mean as in the reference population.

Patients with a lower performance status might deny the participation in the study because they were feeling not well. A second reason might be a bias insofar as elderly patients with comorbidities and bad physical function are not considered for surgery but for conservative treatment [1], [4]. Furthermore, the study center is a tertiary university hospital with patients from all parts of Germany, which aggravate the selection of physical fit patients.

Sustainment if not improvement of quality of life is a main goal of cancer surgery next to survival. Three months after surgery there was a decrease in all functional scales and an increase in symptom burden. Impact of onco-surgery followed by chemotherapy seems to be responsible [9], [52]. Global health status and emotional functioning improved over time and reached the level of the baseline measurement one year after surgery. The symptom burden for the domain appetite loss and nausea and vomiting decreased and reached baseline levels after 12 months. These findings are concordant with previous published studies [9], [56].

Role functioning was significant worse than baseline at the 12 months follow-up. Worse perception of body image due to stoma, abdominal scars, and sexual problems are possible reasons [9].

At one year follow up, the sub domains physical, cognitive and social functioning had not reached baseline level. Several cycles of chemotherapy often follow surgery for cancer and could impair cognitive function [45], [46]. As mentioned above, elderly patients are more vulnerable for postoperative delirium and postoperative cognitive dysfunction (POCD) than younger patients. POCD could be present even 12 months after surgery and longer [48], resulting in difficulties to concentrate or forgetfulness. Braun et al. found improved cognitive function within three months after start of treatment was associated with better survival [44]. We found no association between cognitive function decline and survival. This might be due to the small size of the trial [44].

Fatigue and pain worsened after surgery and improved over time but stayed higher than in the baseline data. Reasons might be chemotherapy as well as advance in the disease [9], [57].

### Limitations

Despite the prospective nature of this study, there are several limitations. The cohort of patients was heterogeneous regarding cancer site, imposing difficulties in the evaluation of disease-specific results. Weakness of statistical power might be attributed to the small number of patients enrolled. This is due to the pilot status of this study.

Without an accurate definition of cause of death, the primary end point was death from all causes. Due to the higher age of the patients with more comorbidities than younger ones, causes of deaths could be age-related. On the other hand, there were no big age differences and no significant differences in comorbidity between survivors and non-survivors.

The EORTC 30 is not specially validated for elderly patients but the EORTC 15 for elderly patients was not available when we conducted the study [3].

As a consequence of this pilot study in the main study nutritional assessment, assessment of body composition and screening for delirium as well as assessment of postoperative cognitive dysfunction were included. Furthermore, gender-related outcome will be addressed, as the power in this study was insufficient.

## Conclusions

The results of our study support that radical onco-surgery is feasible in elderly patients. There is no need to withhold state-of-the-art therapy for fit older patients as still described in the literature [4]. Nevertheless, it seems to be important to identify those elderly patients who are at risk for increased long-term mortality. Patients with cognitive or functional impairment might need further geriatric assessment and tailored intervention to improve outcome after surgery and reducing postoperative morbidity and mortality.

Quality of life measurements prior to surgery may help to identify those areas that have been affected by the disease or by its treatment and could be helpful for the decision for or against major surgery [1], [58].
